# Template-Based Modeling of Protein-RNA Interactions

**DOI:** 10.1371/journal.pcbi.1005120

**Published:** 2016-09-23

**Authors:** Jinfang Zheng, Petras J. Kundrotas, Ilya A. Vakser, Shiyong Liu

**Affiliations:** 1 School of Physics and Key Laboratory of Molecular Biophysics of the Ministry of Education, Huazhong University of Science and Technology, Wuhan, Hubei, China; 2 Center for Computational Biology and Department of Molecular Biosciences, The University of Kansas, Lawrence, Kansas, United States of America; University of Wurzburg, Biocentre, GERMANY

## Abstract

Protein-RNA complexes formed by specific recognition between RNA and RNA-binding proteins play an important role in biological processes. More than a thousand of such proteins in human are curated and many novel RNA-binding proteins are to be discovered. Due to limitations of experimental approaches, computational techniques are needed for characterization of protein-RNA interactions. Although much progress has been made, adequate methodologies reliably providing atomic resolution structural details are still lacking. Although protein-RNA free docking approaches proved to be useful, in general, the template-based approaches provide higher quality of predictions. Templates are key to building a high quality model. Sequence/structure relationships were studied based on a representative set of binary protein-RNA complexes from PDB. Several approaches were tested for pairwise target/template alignment. The analysis revealed a transition point between random and correct binding modes. The results showed that structural alignment is better than sequence alignment in identifying good templates, suitable for generating protein-RNA complexes close to the native structure, and outperforms free docking, successfully predicting complexes where the free docking fails, including cases of significant conformational change upon binding. A template-based protein-RNA interaction modeling protocol PRIME was developed and benchmarked on a representative set of complexes.

## Author Summary

Structures of protein-RNA complexes are important for characterization of biological processes. The number of experimentally determined protein-RNA complexes is limited. Thus modeling of these complexes is important. Reliable structural predictions of proteins and their complexes are provided by comparative modeling, which takes advantage of similar complexes with experimentally determined structures. Thus, in the case of protein-RNA complexes, it is important to determine if similar proteins and RNAs bind in a similar way. We show that, similarly to the earlier published results on protein-protein complexes, such correlation of the protein-RNA binding mode and the monomers similarity indeed exists, and is stronger when the similarity is determined by structure rather than sequence alignment. The data shows clear transition from random to similar binding mode with the increase of the structural similarity of the monomers. On the basis of the results we designed and implemented a predictive tool, which should be useful for the biological community interested in modeling of protein-RNA interactions.

## Introduction

About three quarters of the human genome could be transcribed into RNA, including 4,693 miRNAs [1] and 105,255 long noncoding RNAs [2]. The function of most of these RNAs is unknown. RNAs never act alone. One hypothesis is that the long noncoding RNA are molecular scaffolds for protein binding [3, 4]. Several hundreds of novel RNA-binding proteins (RBP) were discovered by high-throughput sequencing [5, 6]. Protein-RNA complexes play an important role in gene regulation, mRNA degradation and many other biological processes. High-throughput experimental techniques (HITS-CLIP [7], PAR-clip [8], RIP-chip [9]) and computational methods [10–18] have been developed to characterize protein-RNA interactome. These methods identify and characterize protein-RNA interactions, but do not provide the structure of protein-RNA complexes, which is important for understanding the molecular function. An increasing number of experimentally determined protein-RNA structures in PDB are still a fraction of all identified protein-RNA interactions, due to the inherent limitations of the experimental techniques. Thus this gap has to be filled by computational approaches [19].

The principles of protein-RNA interaction are based on structural and physicochemical complementarity [20–22], and are similar to those of protein-protein interactions [23]. Thus the fundamental paradigms of structure prediction should be similar as well: free docking, for protein-protein [23] and protein-RNA complexes [24–29], and the template-based docking, for protein-protein [30] and protein-RNA complexes (investigated in this report). The accuracy of the template-based models is determined by the quality of the selected template, identified by sequence or structure alignment. Whereas the template-based paradigm in protein-protein modeling has been extensively studied and systematically validated/benchmarked [31], similar investigation of template-based approach to protein-RNA complex structure prediction is still lacking (although the approach has been applied to predicting RNA binding sites on proteins in SPOT-Struct-RNA[18]).

We performed such investigation on a representative set of protein-RNA complexes. The analysis of all-to-all alignments in the set revealed a transition point between random and correct binding modes. The results showed that structural alignment significantly outperforms sequence alignment in identifying good templates, suitable for generating protein-RNA complexes with the ligand RMSD from the native structure < 10 Å. A template-based protein-RNA modeling protocol was developed and benchmarked on a representative set of complexes. The study provides a way for protein-RNA structure modeling on a genome scale.

## Methods

### Protein-RNA interaction sets

Co-crystallized protein-RNA structures were downloaded from PDB (1,619 complexes in 2014-05-13 release). Structures with resolution better than 3.0 Å were retained. Multimeric complexes were split into binary ones, defined as one protein chain and one RNA chain. The minimal lengths of the protein and the RNA were 30 and 20 residues, respectively. The interface was defined by < 5 Å distance between any heavy atom of the protein and any heavy atom of the RNA. The minimal numbers of protein and RNA residues at the interface were 5 each. This resulted in 2,951 binary complexes, including 563 RNA chains and 2,721 protein chains. The RNA redundancy was removed by *BLASTClust* [32] with sequence identity cutoff 0.99 and coverage cutoff 0.99. The 563 RNA chains were grouped into 288 clusters. The structure with the highest resolution in a cluster was designated as representative. This resulted in 633 binary complexes, which still included some short identical RNAs due to limitation in the default word size for nucleotides in *BLASTClust*. Thus *CD-hit* package [33] was used to further filter the RNA chains with sequence identity cutoff 0.99. Finally, 439 non-redundant binary complexes (NRBC439) were kept for all-to-all alignment and benchmarking. To determine the predictive power of our program, we split the NRBC439 set into two parts: 80% with an older deposit date were designated as the templates (NRBC349), and 20% with a newer deposit date were designated as targets (*bound set*, NRBC90).

The performance of the template-based and free docking was also tested on the protein-RNA docking benchmark set [34]. To avoid modeling of targets on themselves, 26 complexes that were also part of the template set were excluded. Since in our implementation the template-based protocol can deal only with single-chain proteins and RNAs, the benchmark set was restricted to complexes with single-chain monomers. The length of the RNA chain was ≥ 10 nt according to the alignment procedure (SARA [35]). Although the minimal 20 nt length was used previously [35], in our study successful models were generated with the ≥ 10 nt threshold. The resulting set contained 49 complexes (*unbound set*).

### Target/template alignment

In NRBC439 set all-to-all pairwise alignment was performed by three approaches. The first approach was local sequence alignment by *fasta35* with default parameters [36]. Sequence identity of a complex was defined as the smaller sequence identity of the two monomers. The coverage of the complexes alignment was defined as the lowest coverage of the four chains in the two aligned complexes. The second approach was global sequence alignment by *needle* in the EMBOSS package [37], also with the default parameters. The complex sequence identity and the coverage were defined as in the first approach.

The third approach was structural alignment. For the structure alignment of RNA we chose SARA [35], based on the reported performance characteristics [38] and availability. A newer version, SARA-coffee, is a structure-based multiple RNA aligner, which integrates SARA with R-coffee framework. For pairwise alignment, used in our study, the results of SARA-coffee and SARA are the same. For the structure alignment of proteins, we used *TM-align* [39], following our previous studies of protein-protein complexes [31, 40–44]. The output of *TM-align* is TM-score, which varies from 0 for completely dissimilar structures, to 1 for identical structures. The output of *SARA* is a score, which depends on the RNA size. To establish a similar description of structural similarity of proteins and RNAs, the SARA score was normalized by the score value of the RNA aligned to itself, resulting in the score interval 0–1, similar to the protein alignment. As with the complex sequence identity, the complex structural score was defined as the minimum of TM-score and the normalized SARA score. The aligned atoms (C^α^ in protein and C3' in RNA) were used to calculate interaction RMSD (IRMSD) similarly to the one proposed for protein-protein complexes [45], which numerically characterizes binding mode similarity of complexes of different monomers. It was shown previously to correlate well with the traditional ligand and interface RMSDs for complexes of same monomers in different binding modes (cannot be applied to the complexes of different monomers) [31].

The three alignment approaches were applied to NRBC439 to test the ability to detect a good template. Binary complexes in NRBC90 were queries for the template set NRBC349.

### Building and evaluating models

After a template was selected, the target protein was superimposed on the template protein by *TM-align* and the transformation matrix was saved. The target RNA was superimposed on the template RNA by *SARA*. Since *SARA* does not output the transformation matrix, it was reproduced by superimposing the RNA from *SARA*'s output onto the original query RNA.

The ligand RMSD (RMSD of RNA C3' atoms) between the model and the native structure was calculated. The quality of the model was measured by the ligand RMSD. In protein-RNA docking, a prediction was defined as "acceptable" [28] (elsewhere called "native-like" [26, 29]) for the ligand RMSD ≤ 10 Å from the native structure of the complex, and a more accurate "medium" for the ligand RMSD ≤ 5 Å. These definitions correlate with the ones in protein-protein docking field [46], and the corresponding docking models are generally considered within the intermolecular energy funnel [47] and thus subject to refinement by local optimization.

## Results and Discussion

### Binding mode similarity correlates with the similarity of the monomers

A previous study on template-based protein-protein docking determined strong dependence of the binding mode similarity on the structural similarity of the participating proteins, with the phase transition from dissimilar modes to the similar ones at TMm = 0.4 [31]. In the current study we asked a question: do protein-RNA complexes behave in a similar way? We performed all-to-all pairwise comparison of protein-RNA binary complexes in NRBC439 set. The similarity of the monomers was measured by the sequence alignment (*fasta35* and *needle* for local and global alignment, correspondingly) and by the structure alignments.

**Fig 1:**
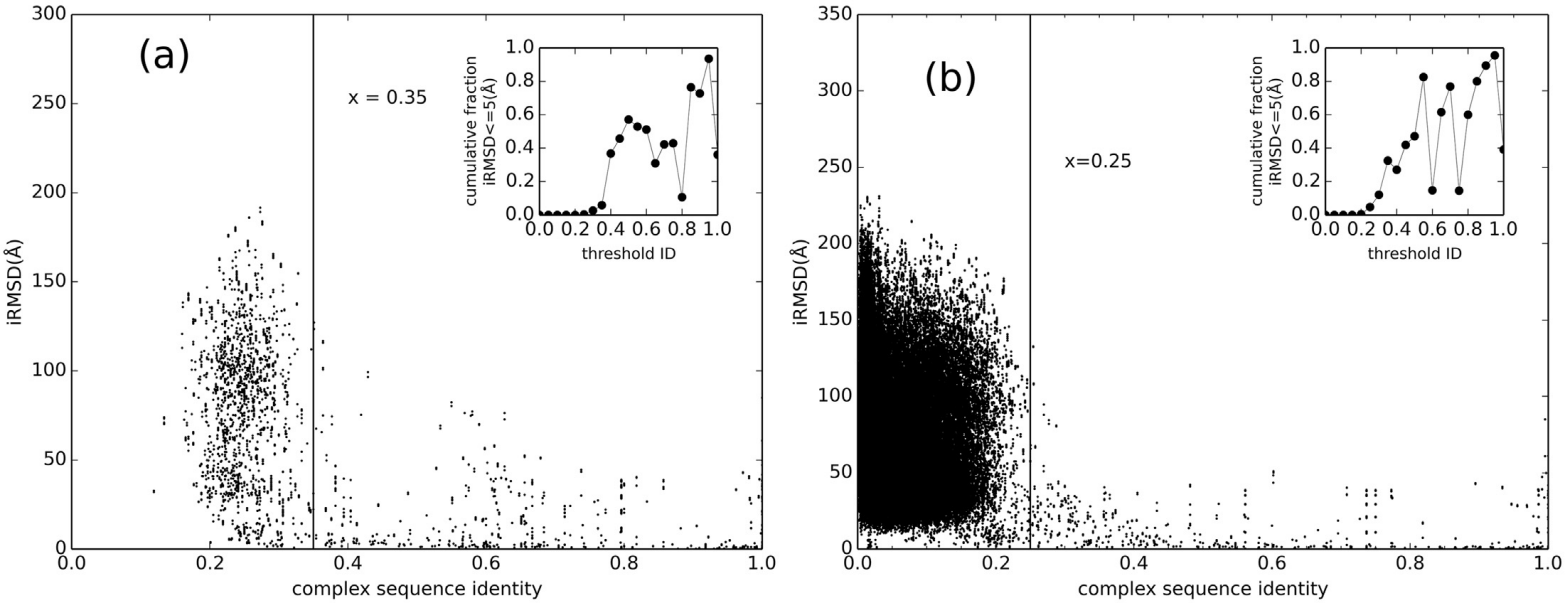
Binding modes vs. sequence identity. For local (a) and global (b) sequence alignments, IRMSD is plotted against the complex sequence identity (the smallest of the monomers sequence identity), in all-to-all pairwise comparison of 439 binary complexes. The data for the local alignment is restricted to alignments with coverage ≥ 0.3 (see text). The insets show the fraction of complex pairs with IRMSD ≤ 5 Å plotted in 0.05 bins to show the phase transition, indicated by the vertical lines on the main plot.

Fig 1 shows the results of such comparison for local and global sequence alignments. For the local alignment, the 0.3 coverage threshold is used. The 0.3 value was the optimal, minimizing the noise from the lower threshold alignments (results with no threshold for the coverage were largely random), while retaining 420 of 438 binary complexes for the analysis. The dip in cumulative fractions near 0.8 threshold value may be random, due to low sampling at this data range. One can also speculate that some of RBPs may have close homologs, with sequence ID near this value, whereas recent analysis showed that most RBPs are more diverse [48].

**Fig 2:**
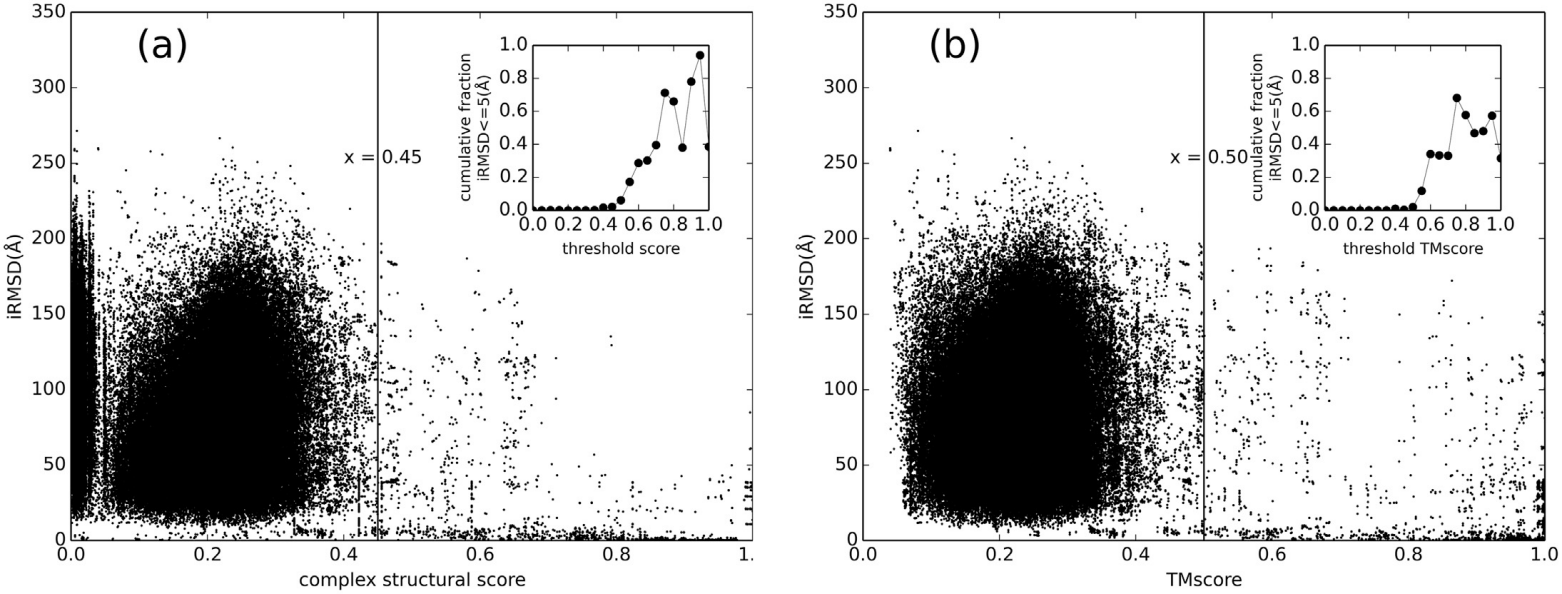
Binding modes vs. structural similarity. IRMSD is plotted against (a) complex structural similarity (the smallest of the TM-score and normalized SARA score) and (b) protein structure similarity, in all-to-all pairwise comparison of 439 binary complexes. The insets show the fraction of complex pairs with IRMSD ≤ 5Å plotted in 0.05 bins to show the phase transition, indicated by the vertical lines in the main plot.

As the figure shows, the transition to similar binding modes occurs near the complex sequence identity 0.3. The results of such comparison obtained by the structure alignment approach are shown in Fig 2a. The transition point on the alignment distributions was used as a cutoff for selecting good templates. S1 Fig shows that the success rate of detecting templates begins to decrease near the transition point (complex structural score 0.45).

To distinguish the role of the protein in detecting a good template for a protein-RNA complex, the target/template similarity was also measured only for the protein component (Fig 2b). This distribution is similar to the one in Fig 2a, indicating an important role of the protein. However, the role of the RNA is evident at the higher end of the structural similarity (> 0.7), where it eliminates multiple alternative binding modes. Thus the similarities of both protein and RNA are needed for an accurate identification of a good template for the complex. Overall, correlation of the protein-RNA structural similarity with the binding mode is weaker than that of the protein-protein complexes [31] because of the greater RNA flexibility [49, 50].

### Comparison of sequence and structure similarity

**Fig 3:**
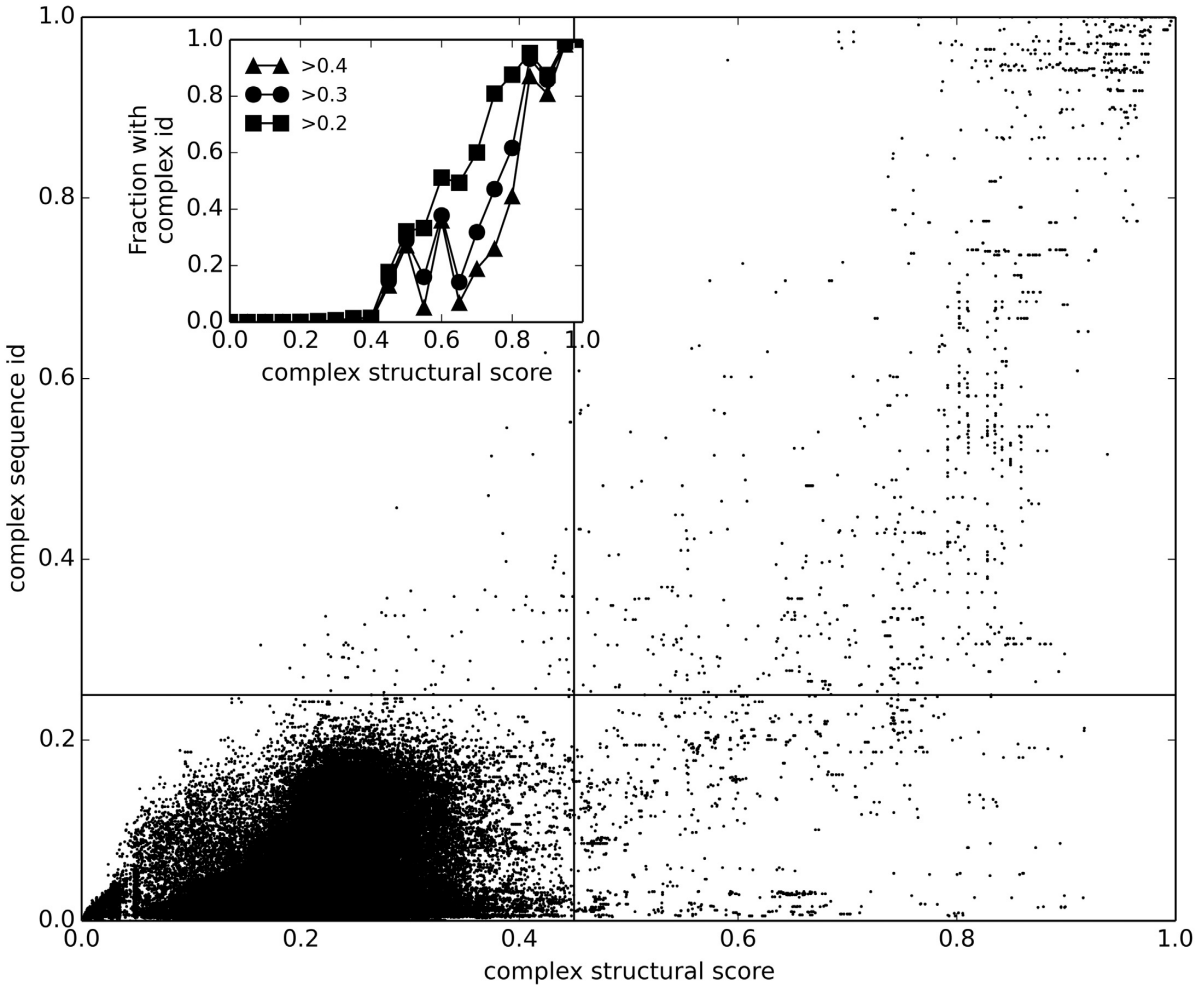
Structural vs. sequence similarity of protein-RNA complexes. The structural similarity of the complexes is plotted against the sequence identity in all-to-all pairwise comparison of 439 binary complexes. The lines separate quadrants below and above a sequence and a structure-based threshold (see text). In the inset, the fraction of the binary complex pairs with the complex sequence identity > 0.4, 0.3 and 0.2 is plotted in 0.05 bins of complex structural score, showing that many pairs with a similar structure have low sequence identity < 0.4, 0.3 or 0.2.

Structural similarity vs. sequence identity of the protein-RNA complexes is plotted in Fig 3. The plot is divided into four areas by the lines x = 0.45 (transition point for structural similarity), and y = 0.25 (transition point for sequence similarity). The correlation of structure and sequence similarity in protein-RNA is similar to that in protein-protein complexes [31]. The structure and sequence are dissimilar in the lower left quadrant, which contains 98.4% of the alignments. This points to the diversity of sequences and structures in NRBC439 set (supported by observation that 1,542 RBPs formed 1,111 families in human RBPome [48]). The upper right quadrant contains 1.02% of the alignments, and 69.53% of those with the structural score ≥ 0.45, where structure and sequence are similar, suggesting that both approaches can find a good template. The alignments with similar structure and dissimilar sequence are in the lower right quadrant, containing 0.45% of alignments, and 30.47% of those with the structural score ≥ 0.45. This suggests that structural alignment approach could find good templates for about 1/3 of cases when sequence alignment cannot. Last, the top left quadrant shows similarity detected by the sequence, but not the structural alignment. It is almost empty, which means that the structural alignment finds most templates detectable from the sequence.

### Benchmarking of docking

**Fig 4:**
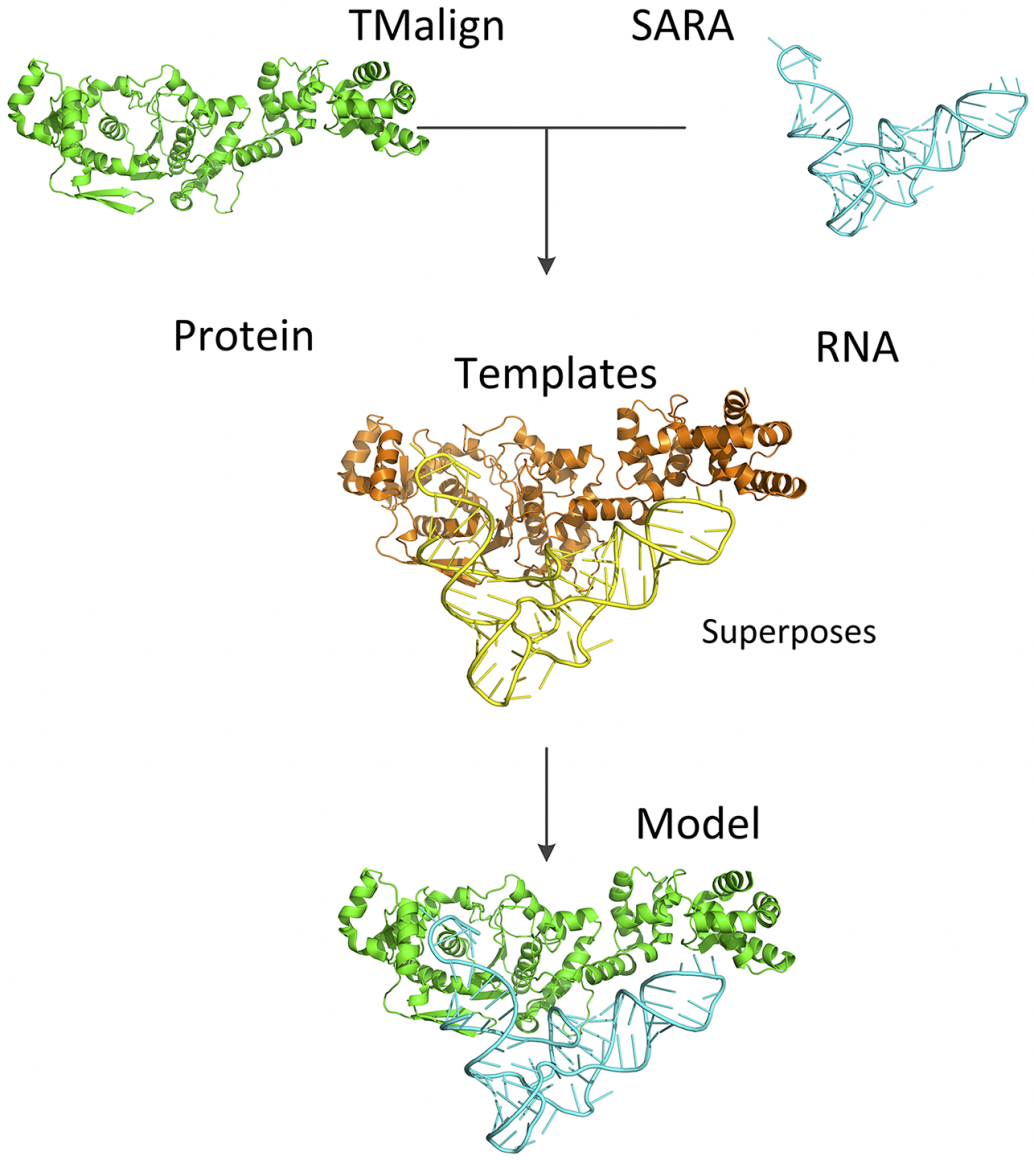
Protein-RNA modeling procedure. The input protein and RNA structures are aligned to the templates by *TM-align* and *SARA* correspondingly. The models of the complex are sorted by the complex structural score (see text).

**Fig 5:**
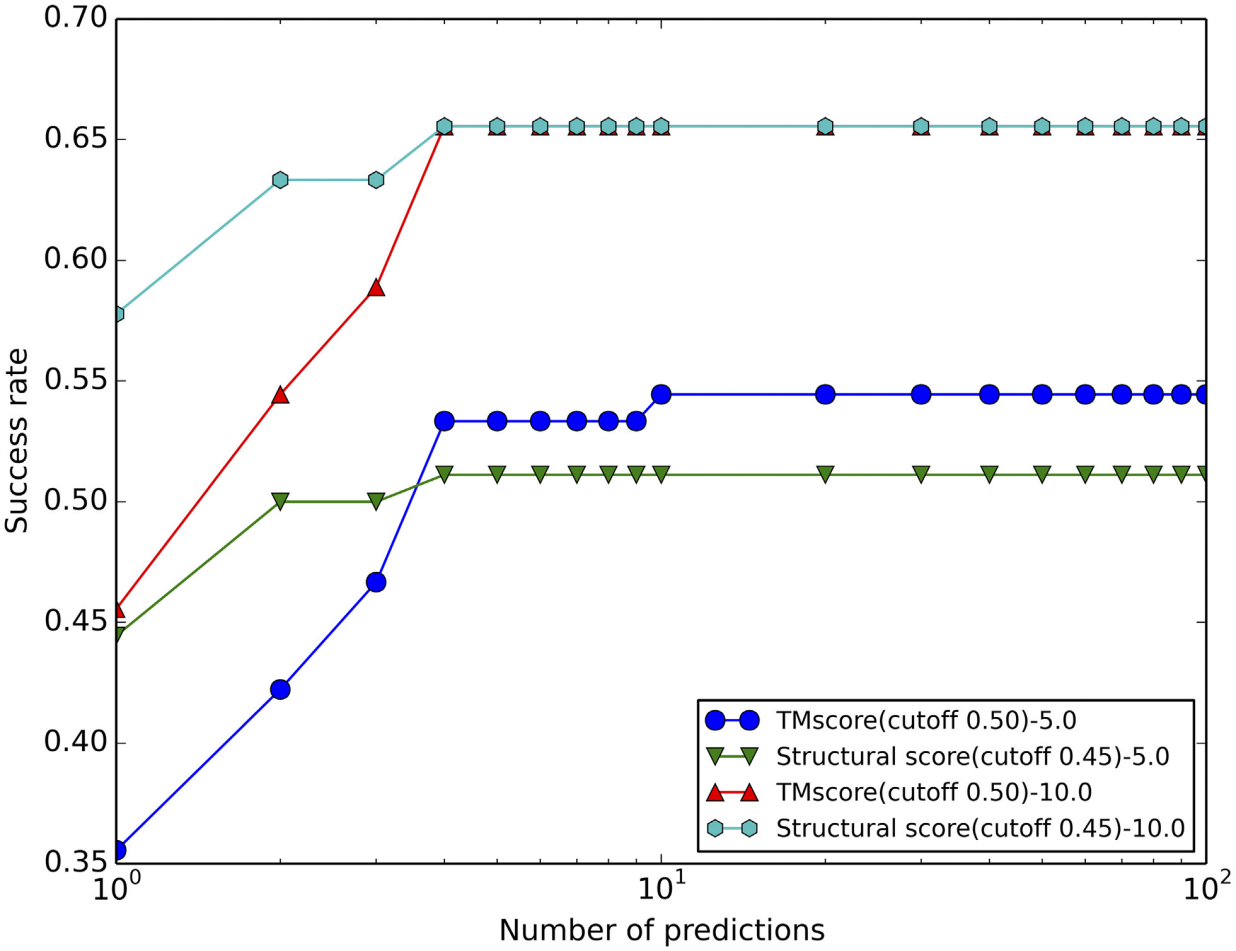
Benchmarking of template-based protein-RNA structure prediction. Targets (90 newer complexes) were predicted using templates (349 older complexes). The models are ranked separately by the complex structural score and by the TM-score. The docking of a complex was successful if at least one prediction within a set number of predictions was successful (RMSD between predicted and native structures ≤ 5 Å for "medium" and ≤ 10 Å for "acceptable", see Methods). Score with cutoff X means that the model is built from the template with a target/template score larger than the transition point X.

A structure alignment-based docking was implemented in a procedure PRIME (Protein-RNA Interaction ModEling). Fig 4 shows the outline of the approach. Docking was systematically benchmarked on NRBC90 targets using NRBC349 templates. For each target docking models were generated by PRIME, ranked separately by the complex structural score and by the TM-score. The success rates of different approaches are shown in Fig 5. The success rate for predicting "acceptable" model almost reaches the highest value after top 4. This suggests that for the docking, the complex structural score, which accounts for both TM-score for proteins and SARA score for RNA, is better than just the TM-score for proteins in top 1, top 2, and top 3. The TM-score outperformed or tied with the complex structural score when considering more top models. The TM-score detected the template for three complexes, for which the complex structural score could not. The reason was that when the normalized SARA score was counted in, the complex structural score decreased below the cutoff (score values 0.37, 0.016, and 0.15). The improvement of the success rates for top 1, top 2, and top 3 predictions with the complex structural score was largely due to the reduction of noise after the transition point in Fig 2 (by moving it to the left of the transition point). For example, the alignment of the target complex 3umy, chains A and B, and the template complex 2hw8, chains A and B, had IRMSD = 28.04 Å, but the TM-score 0.90, ranked 2 by the TM-score alone. At the same time, the corresponding complex structural score is 0.29, ranked 51, moving the complex to the left of the transition point, and thus reducing the noise for the high scored complexes.

**Fig 6:**
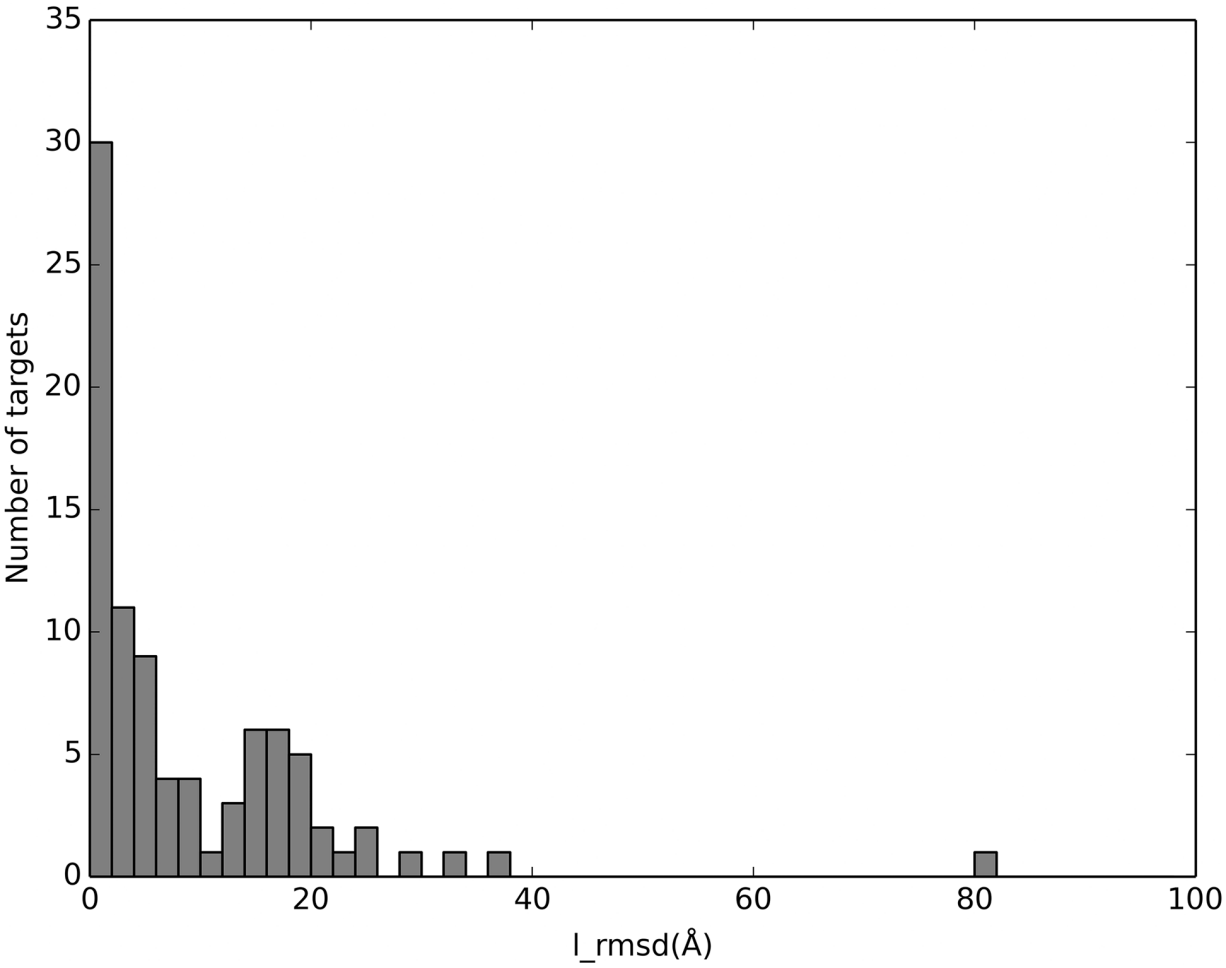
Distribution of best models of the complexes according to the ligand RMSD.

Fig 6 shows the distribution of the best models according to ligand RMSD. The distribution is bimodal, pointing to the existence of alternative binding modes, similar to protein-protein complexes [31]. The high-quality predictions (0–2 Å) correspond to 30 targets (33%).

Benchmarking of PRIME suggests that 65% of target models can be built successfully (structural score-10.0 for top 10 predictions in Fig 5). Ranked by the complex structural score, most models with "acceptable" quality are ranked at top 4. Similar to protein-protein modeling, the template-based protein-RNA docking has a clear advantage over the free docking method, where scoring functions typically are struggling to pick the correct model from the multitude of docking poses [29]. The template-based method of course cannot be applied when a template is not found, in which case the free docking should be used. In our benchmark, templates were detected for 69 out of 90 targets.

**Fig 7:**
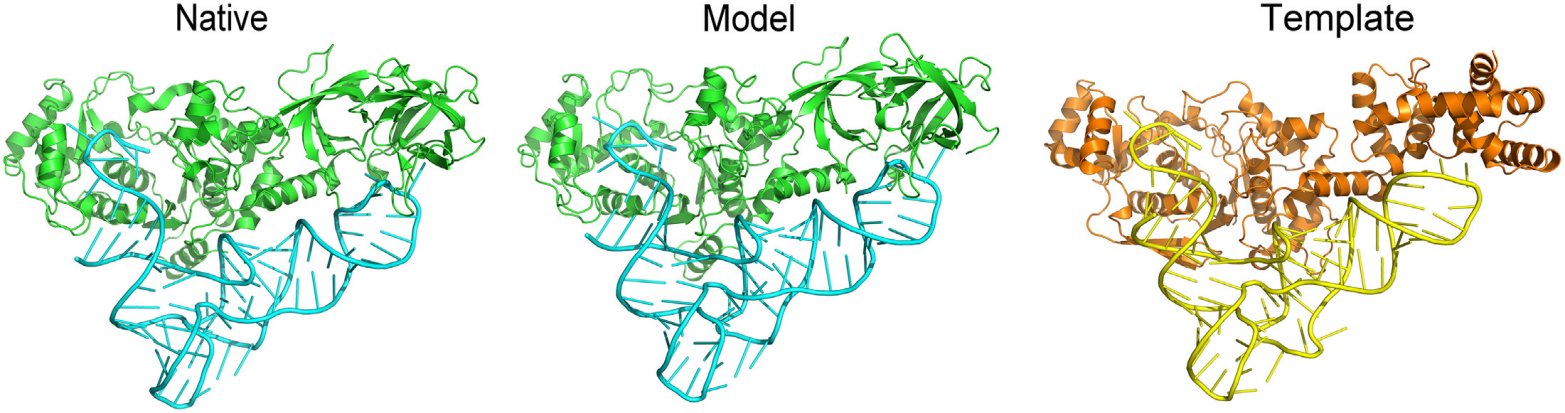
An example of a target modeled by structure alignment. The target 1euy, chains A and B[57], was modeled on the template 1n78, chains A and C[58]. The target/template sequence identity for the protein is 0.20 and RNA is 0.52, which is relatively low for the protein. The structural similarity is high: TM-score 0.57 for the protein and the normalized SARA score 0.78 for the RNA. The ligand RMSD for the model is 3.46 Å.

Fig 7 shows an example of the target with low protein sequence identity to the template, successfully modeled by the structure alignment. Still, structure similarity does not guarantee correct predictions. The alternative binding modes were observed in nine targets with high structural similarity to the templates. Although the complex structural scores of their alignment to the templates were larger than the transition point, the ligand RMSD of the models built on these templates were > 10 Å. For example, the TM-score, normalized SARA score and the complex structural score between the target 4lgt, chains A and E, and the template 2i82, chains A and E, were 0.543, 0.524 and 0.524, respectively. However, the binding mode is different, with the model/native ligand RMSD 22.45 Å.

### Comparison of template-based and free docking

**Fig 8:**
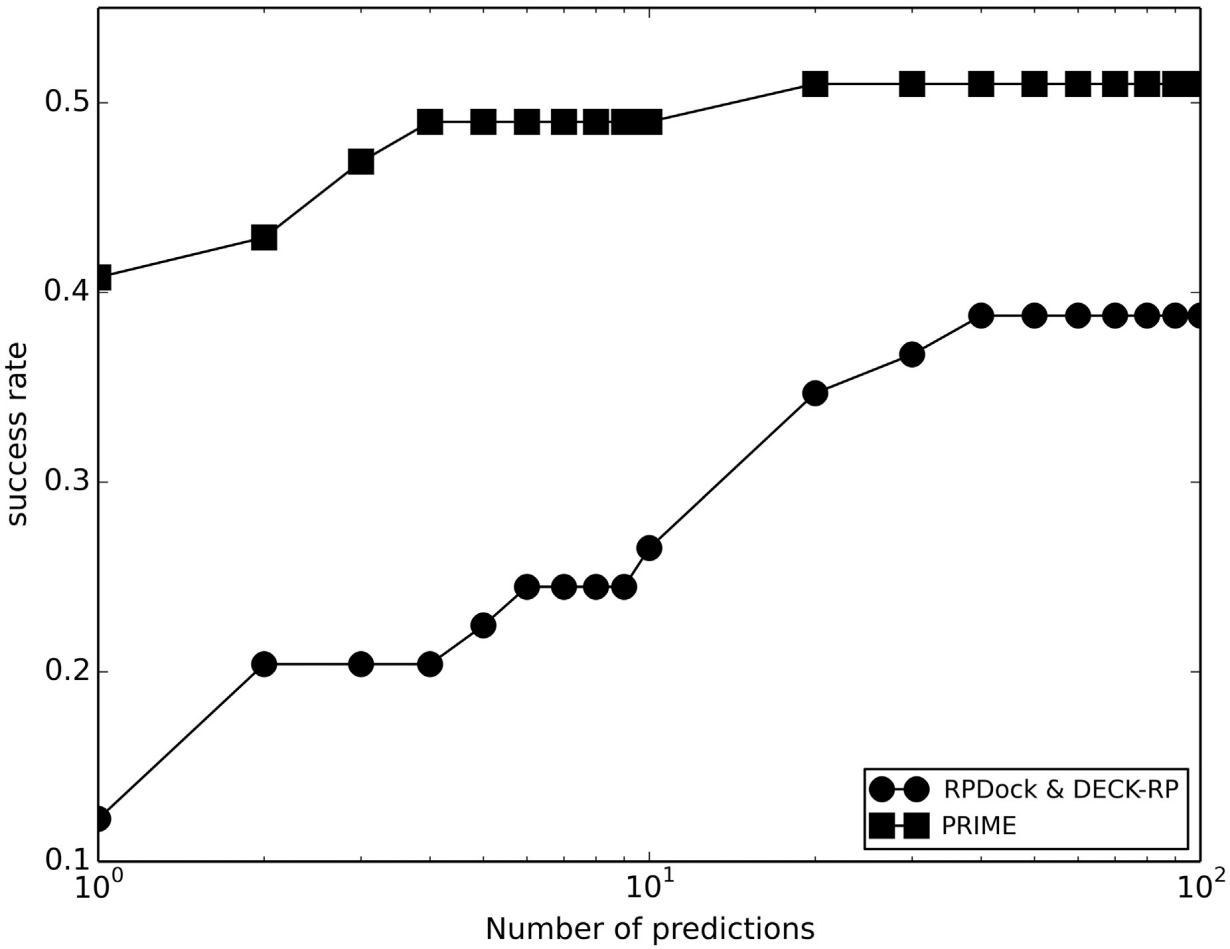
Comparison of the template-based and free docking. The template-based docking was performed by PRIME, and the free docking by RPDock. The successful prediction was defined as the one with at least one match with ligand RMSD ≤ 10 Å in top *N* predictions.

To compare the performance of template-base and free docking method, we tested template-based PRIME and free docking RPDock on the unbound set (see Methods). RPDock [29] is a protein-RNA rigid docking protocol, which takes into account protein/RNA geometric and electrostatic complementarity, and stacking interaction in the base of nucleotides with the aromatic rings of charged amino acids. All PRIME models were ranked by the complex structural score, and RPDock models were ranked by DECR-RP [29]. Fig 8 shows the docking results. Success rate is defined the number of those with at least one "acceptable" model divided by the total number of targets. The results show that the success rate of the template-based protein-RNA docking is significantly higher than that of the free docking, similarly to the previous results in protein-protein docking [41] (although a broader assessment of the protein-protein category is still on-going [51, 52]). The detailed data on benchmarking (S1 Table) indicates that the template-based approach significantly outperforms free docking, successfully predicting complexes where the free docking fails, including cases of larger bound/unbound RMSD (see S2 Table, and an example of a successful template-based prediction of a complex with a significant conformational change on the protein component in S3 Fig). PRIME also runs ~ 5 times faster than RPDock (S2 Fig), which is especially important for genome-scale studies.

PRIME currently does not include a refinement protocol, which is still a challenging task in macromolecular docking [46]. The development of a dedicated refinement protocol is in our future plans. However, even a standard minimization by GROMACS (v5.0.7) [53]with AMBER99 force field reduced the number of clashes in most complexes (S4 Fig).

### Conclusion

Sequence and structure alignment approaches were compared in template-based modeling of protein-RNA complexes. All-to-all alignment of protein-RNA complexes detected a phase transition from random to similar binding modes, according to the degree of monomers similarity. The structure alignment showed to be significantly better than the sequence alignment in identifying correct templates. In systematic benchmarking, structure alignment-based docking had far better success rate than the free docking, successfully predicting complexes where the free docking failed, including interactions with significant conformational change upon binding. The findings are qualitatively similar to those observed earlier in structural modeling of protein-protein complexes [31]. Applicability of the prediction protocols to complexes of modeled monomers, rather than to experimentally determined structures of monomers, which typically have higher accuracy than models, was previously established for protein-protein interactions in systematic benchmarking studies on specifically designed sets of protein models[54, 55]. Similar studies are needed to determine such applicability to modeled RNAs [56]. The structure alignment-based approach for protein-RNA modeling is implemented in PRIME software, publicly available at http://rnabinding.com/PRIME.html.

## Supporting Information

S1 Fig Detection of templates at different structure similarity thresholds. (PDF)

S2 Fig Comparison of PRIME and RPDock computation time. (PDF)

S3 Fig A model with a large unbound/bound conformational change. (PDF)

S4 Fig Clashes before and after refinement. (PDF)

S1 Table PRIME and RPDock benchmarking on protein-RNA set. (XLSX)

S2 Table Number of successfully docked benchmark complexes. (PDF)
