# Supplementary material for: Template-Based Modeling of Protein-RNA Interactions
Source: PLoS Comput Biol. 2016 Sep 23;12(9):e1005120. doi: 10.1371/journal.pcbi.1005120 (PMC5035060; doi:10.1371/journal.pcbi.1005120)
Supplement: S1 Fig — (PDF) [file pcbi.1005120.s001.pdf]

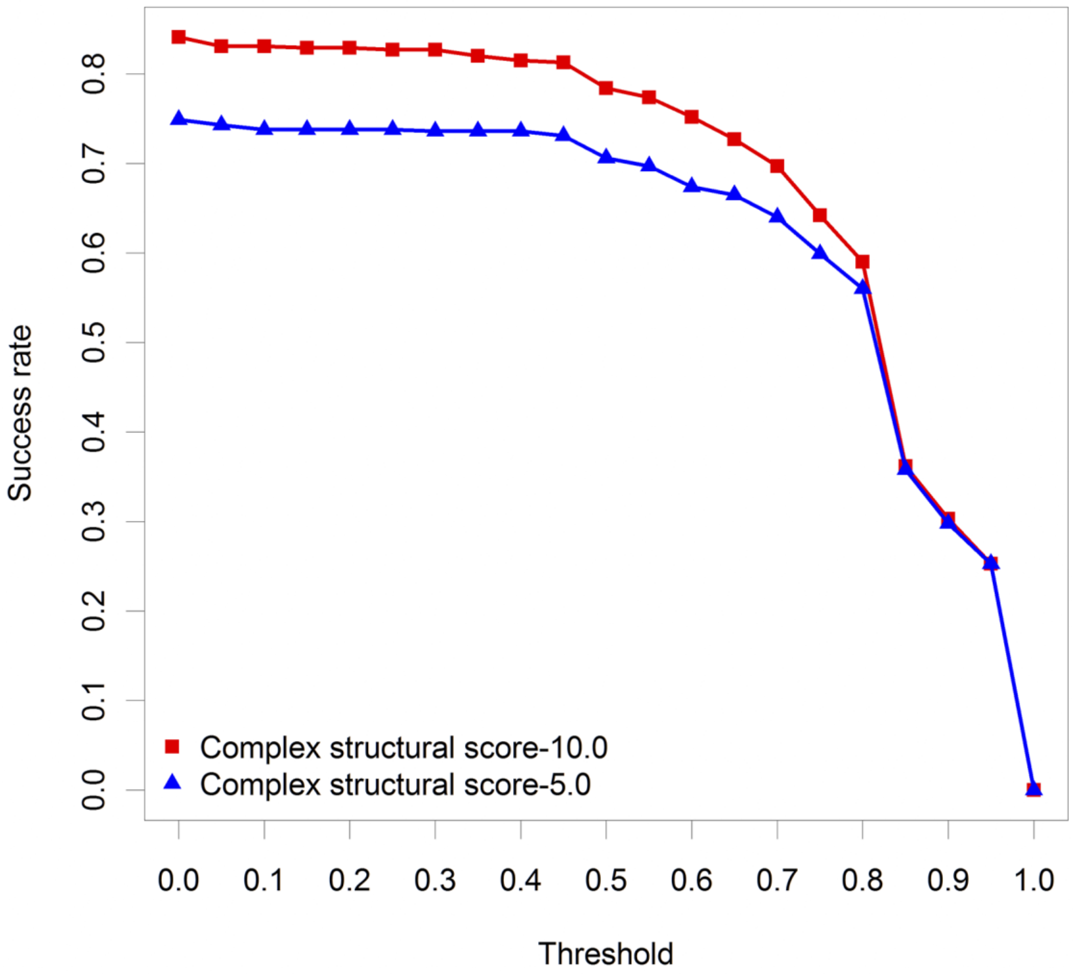

S1 Figure. *Detection of templates at different structure similarity thresholds*. The results of all-to-all comparison of complexes in NRBC439 set (see main text) confirm the transition that occurs near the threshold value 0.45. Success rate was defined as the IRMSD < 5 or 10 Å ("complex structural score" 5 or 10).
