# Supplementary material for: Template-Based Modeling of Protein-RNA Interactions
Source: PLoS Comput Biol. 2016 Sep 23;12(9):e1005120. doi: 10.1371/journal.pcbi.1005120 (PMC5035060; doi:10.1371/journal.pcbi.1005120)
Supplement: S2 Fig — (PDF) [file pcbi.1005120.s002.pdf]

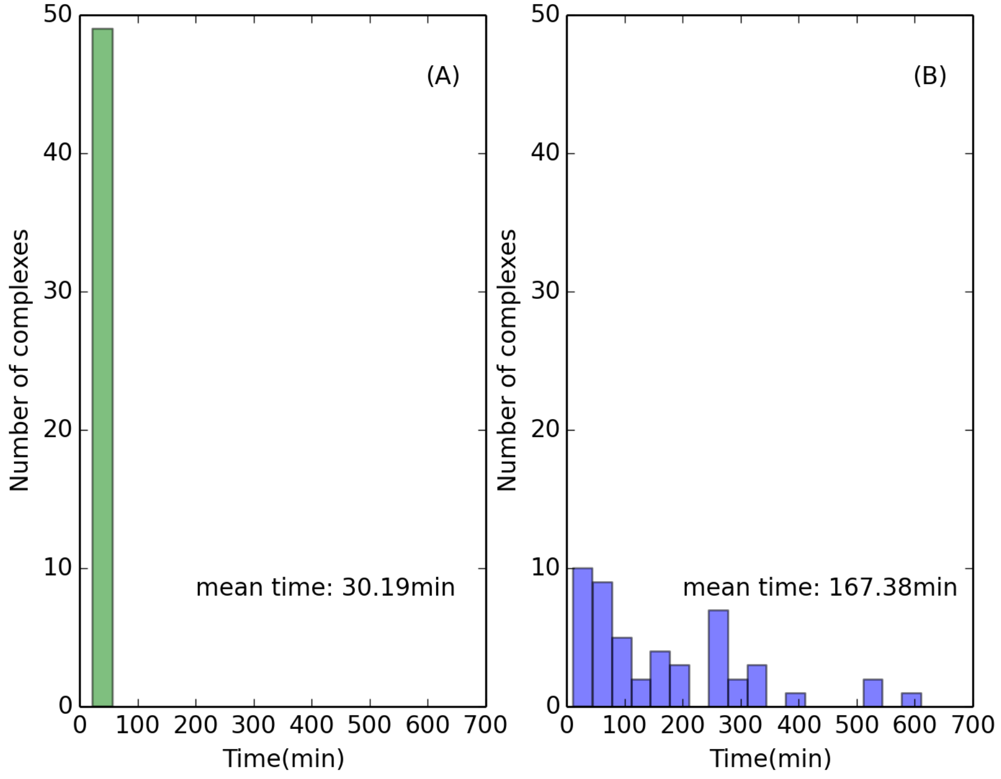

S2 Figure. *Comparison of PRIME and RPDock computation time*. The protein-RNA benchmark set (see main text) was modeled by PRIME (A) and RPDock (B) on Intel X5650 with 24GB RAM, showing that PRIME is ~ 5 times faster than RPDock.
