# Supplementary material for: Template-Based Modeling of Protein-RNA Interactions
Source: PLoS Comput Biol. 2016 Sep 23;12(9):e1005120. doi: 10.1371/journal.pcbi.1005120 (PMC5035060; doi:10.1371/journal.pcbi.1005120)
Supplement: S3 Fig — (PDF) [file pcbi.1005120.s003.pdf]

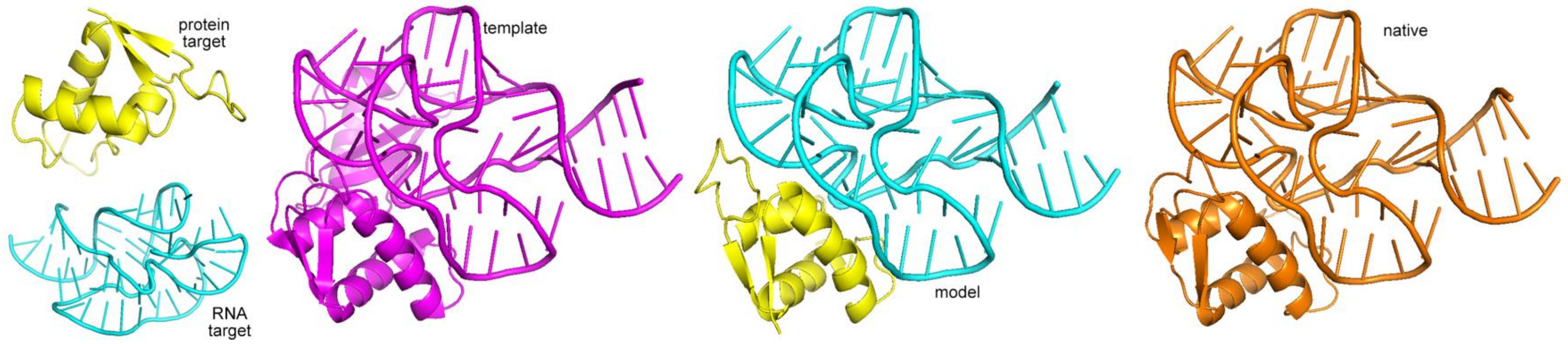

S3 Figure. *A model with a large unbound/bound conformational change.* The target (protein 1xpi chain A, and RNA 2i82 chain E) is modeled on the template 2ab4 chains A and B) by PRIME. The ligand RMSD between the model and the native complex 2i82 chains A and E is 5.7 Å. Free docking RPDock did not model the target successfully due to the large conformational change.
