# Supplementary material for: Template-Based Modeling of Protein-RNA Interactions
Source: PLoS Comput Biol. 2016 Sep 23;12(9):e1005120. doi: 10.1371/journal.pcbi.1005120 (PMC5035060; doi:10.1371/journal.pcbi.1005120)
Supplement: S4 Fig — (PDF) [file pcbi.1005120.s004.pdf]

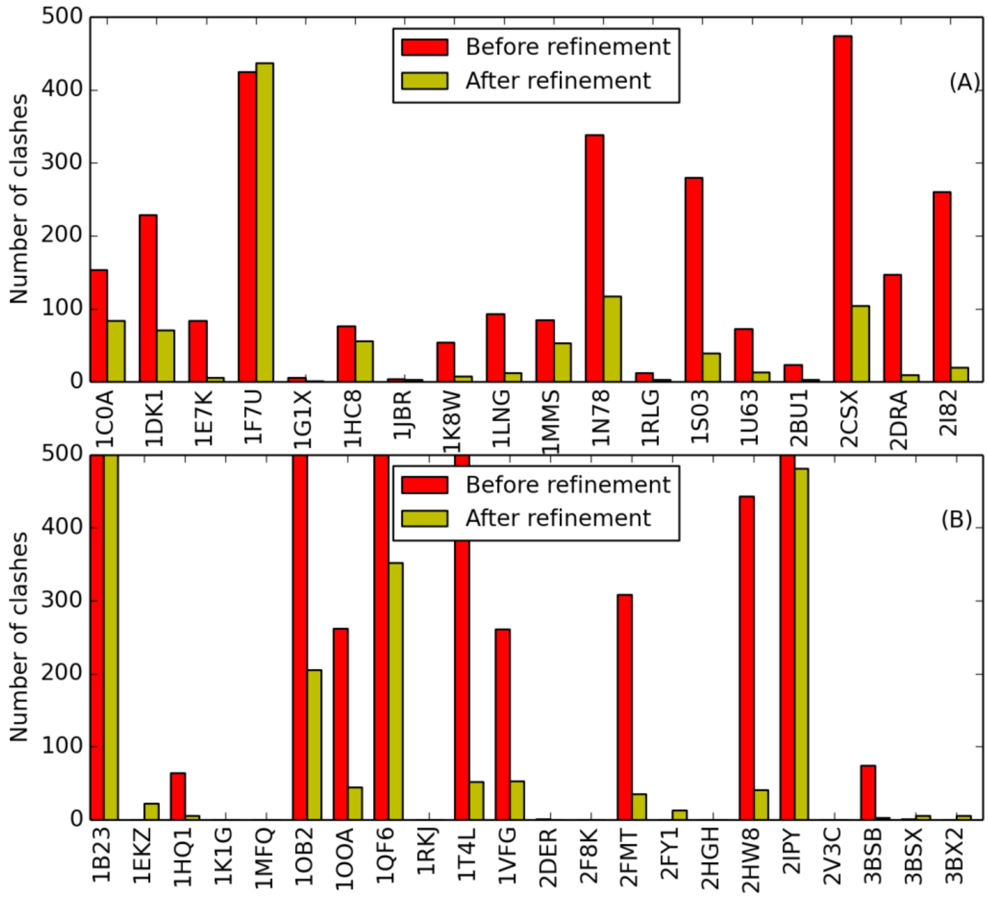

S4 Figure. *Clashes before and after refinement*. Clashes are in the first hit in top 4 models of successfully predicted complexes (A), and in the top 1 model in unsuccessfully predicted complexes. The energy was minimized by GROMACS (v5.0.7) with AMBER99 force field. Nine out of 49 targets failed minimization because of the large structural overlaps. In the successfully predicted targets, the number of clashes is reduced in 17 out of 18 models, and slightly increases for one (1f7u). The number of clashes decreases also in the unsuccessfully predicted targets. The results show that the physics-based refinement can remove atomic collisions in modeled protein-RNA complexes. Development of dedicated refinement procedures should further improve the structural quality of the predictions.
