# Supplementary material for: Template-Based Modeling of Protein-RNA Interactions
Source: PLoS Comput Biol. 2016 Sep 23;12(9):e1005120. doi: 10.1371/journal.pcbi.1005120 (PMC5035060; doi:10.1371/journal.pcbi.1005120)
Supplement: S2 Table — (PDF) [file pcbi.1005120.s006.pdf]

S2 Table. Number of successfully docked benchmark complexes

Conformational change (Å)	0 - 2	2 - 4	4 - 6	6 - 8	8 - 10	10 - 13
Number of complexes	14	13	9	4	2	4
PRIME	9	9	5	1	1	0
RPDock & DECK-RP	6	4	1	2	0	0

Conformational change is max RMSD between bound and unbound structure of RNA or protein

Complex is successfully docked if at least one hit with ligand RMSD <10 Å is in top 100
